# The Voice of Medical Science at Pretoria

**Published:** 1896-05-30

**Authors:** 


### The Hospital

An Institutional Journal of

The Medical Sciences and Hospital Administration, with

AN EXTRA NURSING SUPPLEMENT.

Vol. XX.—No. 505.] [May 30, 1896.

### The Voice of Medical Science at Pretoria.

At the present moment it is imperative that the world should understand what is going on at the Pretoria gaol. In civilised warfare, when the battle is over the doctor comes in. He comes in with universal approval. Men of all nations recognise that those who are wounded in the strife are entitled to be sheltered thereafter in wholesome havens of peace, and to be restored, if science can restore them, to health and working power. Even if one side have been a "rebellious" side, and if its rebelliousness has failed, it is still recognised that to legitimate punishment there must not be added conditions which impair the health, depress the mental powers, and endanger life. All this, we say, is an established part of civilisation, and one which civilisation must do its utmost to maintain.

Now let us consider calmly, in the light of the common principles of civilisation, what is actually taking place at the Pretoria gaol at the present moment. For several months past the gaol surgeon, an official appointed by the Government of the Transvaal, has repeatedly condemned to the authorities the sanitary condition of the gaol. Whether or not his condemnations have been justified let the facts tell. For example, the political prisoners of the Rand are kept in "small and unhealthy sheds." What does Surgeon Messum mean by "small and unhealthy sheds"? He means that each prisoner is allowed 150 cubic feet of space to live and sleep in, and no more. In English prisons from 800 to 1,200 feet are allowed for each prisoner, and that in prisons where, not political prisoners, but the basest scoundrels are immured. In the worst days of the Bastille, and in the filthiest prisons visited by Howard in England and the Continent more than a hundred years ago, it was seldom that such limited space as this was accorded even to the very vilest. These miserable cells at Pretoria are not ventilated; they have not even windows, but only "small holes" in the wall, which let in hardly any fresh air, but only continuous draughts almost more dangerous than the foul air generated in the cells themselves. Imagine life sustained for one year, two years, fifteen years, in places like these by educated men, whose only fault it has been that they have striven by force to obtain some elementary political rights—striven, but failed. Had they succeeded they would have been hailed as heroes.

What are these political prisoners supplied with for beds? "Straw mattresses," and these placed not on bedsteads, but on the "bare soil," which, as the surgeon simply adds, "is very unhealthy in wet weather." No doubt it is. If any gently nurtured man or woman will try to realise the feeling of sleeping on a straw mattress, on the bare ground, in wet weather in a little partitioned-off space in an iron hut of 150 cubic feet capacity, he will feel something more than sympathy with the political prisoners of the Rand, and something like an incipient sensation of indignation against the suzerain Power.

Certain other facts may be mentioned, but we have not space to adequately enforce their significance. For example, such things as urinals have no existence at Pretoria gaol. Vermin of all kinds abound, as may be imagined, in that portion of the prison where the blacks are confined, and the political prisoners are in the "immediate vicinity" of the blacks. New latrines which are being built are not ready for use, and it is impossible to say when they will be ready. "The iron huts," of which part of the prison consists, are so hot by day and so cold by night as to be almost unbearable. Already the consequences are beginning to be serious. To say nothing of poor Grey, who committed suicide, prisoners Buckland, Lingham, and Joel have developed "serious throat affections," and have had to be removed to hospital, although the hospital accommodation itself appears to be little better than that of the prison cells. Prisoner Mosenthal is ill with diabetes, Captain Mein has a lung affection, prisoner Duirs has developed homicidal mania, and it is feared another tragedy may result if he be not removed. Add to all this, that in consequence of the prevailing insanitary abominations an epidemic is threatened, and that the "surgeon's reports are entirely disregarded by the authorities, who are utterly callous," and there is a picture presented to the medical mind which for cold-blooded, unscientific ignorance and uncivilised cruelty could not, we believe, be paralleled anywhere else in a civilised country.

Now this is how the case presents itself to the medical mind; and it is the voice of medicine which is entitled to be heard at the present juncture, and that above all other voices. For, as we have said, the conflict is over, the "rebels" have had the worst of it, and their punishment has been meted out and must be endured. But all that we have described is no part of their punishment. In no other country would they have had it to endure. Is it to be tolerated that a small and ignorant country may do with impunity and because of its ignorance what no other civilised country would venture on any consideration whatever to do at all?

